# Fractal-Based Analysis of fMRI BOLD Signal During Naturalistic Viewing Conditions

**DOI:** 10.3389/fphys.2021.809943

**Published:** 2022-01-11

**Authors:** Olivia Campbell, Tamara Vanderwal, Alexander Mark Weber

**Affiliations:** ^1^School of Biomedical Engineering, University of British Columbia, Vancouver, BC, Canada; ^2^British Columbia (BC) Children's Hospital Research Institute, UBC, Vancouver, BC, Canada; ^3^Department of Psychiatry, University of British Columbia, Vancouver, BC, Canada; ^4^Division of Neurology, Department of Pediatrics, University of British Columbia, Vancouver, BC, Canada; ^5^Department of Neuroscience, University of British Columbia, Vancouver, BC, Canada

**Keywords:** Hurst exponent, movie fMRI, signal dynamics, default network, HCP 7T data, scale invariance, multistability

## Abstract

**Background:** Temporal fractals are characterized by prominent scale-invariance and self-similarity across time scales. Monofractal analysis quantifies this scaling behavior in a single parameter, the Hurst exponent (H). Higher H reflects greater correlation in the signal structure, which is taken as being more fractal. Previous fMRI studies have observed lower H during conventional tasks relative to resting state conditions, and shown that H is negatively correlated with task difficulty and novelty. To date, no study has investigated the fractal dynamics of BOLD signal during naturalistic conditions.

**Methods:** We performed fractal analysis on Human Connectome Project 7T fMRI data (n = 72, 41 females, mean age 29.46 ± 3.76 years) to compare H across movie-watching and rest.

**Results:** In contrast to previous work using conventional tasks, we found higher H values for movie relative to rest (mean difference = 0.014; *p* = 5.279 × 10^−7^; 95% CI [0.009, 0.019]). H was significantly higher in movie than rest in the visual, somatomotor and dorsal attention networks, but was significantly lower during movie in the frontoparietal and default networks. We found no cross-condition differences in test-retest reliability of H. Finally, we found that H of movie-derived stimulus properties (e.g., luminance changes) were fractal whereas H of head motion estimates were non-fractal.

**Conclusions:** Overall, our findings suggest that movie-watching induces fractal signal dynamics. In line with recent work characterizing connectivity-based brain state dynamics during movie-watching, we speculate that these fractal dynamics reflect the configuring and reconfiguring of brain states that occurs during naturalistic processing, and are markedly different than dynamics observed during conventional tasks.

## Highlights

- Fractal analysis of fMRI data reveals differences in temporal signal dynamics between movie-watching and resting state conditions.
- Movie vs. rest differences in fractal dynamics emerge at both the whole-brain level and by resting state networks.
- The Hurst exponent has moderate-strong test-retest reliability in both conditions.

## Introduction

Fractals emerge when the repetition of a simple process creates a recursive structure (Eke et al., 2000), such as the growth of a tree from one branch to two. They are defined by scale-invariance, meaning that the pattern that emerges when the process is repeated remains the same regardless of the scale at which it is viewed. While fractal patterns may be easiest to visualize in the spatial domain, such as in the geometric structure of a snowflake, tree, or perfect fractal, they also exist in the time domain. In temporal fractals, a sequence of events occurs at no dominant time-scale and a recursive structure emerges in the corresponding time-series. As such, temporal fractals are also characterized by self-affinity and power-law behavior (Eke et al., 2002). The former refers to the notion that when a smaller element of the signal is magnified along the time axis it resembles a larger element of the whole. The latter denotes that if you double the frequency, the power diminishes by the same fraction [the spectral index, or Beta (β)] regardless of the chosen frequency on the signal's power spectral density (PSD). More formally, the power law is expressed as |*A*(*f*)|^2^∝*c*·*f*^−β^ on the log-log representation of the PSD, where β is the negative slope of a straight line fitting the distribution (Eke et al., 2000).

Scaling behavior is a pervasive and powerful phenomenon in the universe. Since fractals were first identified in the 1980s (Mandelbrot et al., 1983), they have been recognized in natural objects, physiological systems, and more recently, the brain. In the past two decades, numerous neuroimaging studies have reported scaling behavior in both the spatial domain (i.e., dendritic branching) and time domain (i.e., neuronal activity) (Caserta et al., 1995; Mazzoni et al., 2007; Bédard and Destexhe, 2009). Where spatial fractals can provide information about anatomical neurodevelopment and organization, temporal fractals present an exciting opportunity to explore the signal dynamics that mediate neural functioning over time. Among other functional imaging modalities (Bullmore et al., 1994; Bassett et al., 2006; Moser et al., 2019; Zhu et al., 2020), the temporal scaling patterns in the brain are captured in the fMRI BOLD signal, which measures hemodynamic variation in brain tissue over time (Ogawa et al., 1990). The persistence of fractal properties in the BOLD signal can be quantified with fMRI fractal analysis, which describes the dynamics of the hemodynamic fluctuations in the brain. While other analyses of fMRI data often report topological or connectivity patterns that emerge at a specific scale (He, 2011), fractal analysis is a signal-based method that describes the correlation structure of a process across temporal scales (Eke et al., 2002). Thus, the technique provides novel and complementary information about the structural mechanisms that underlie neuronal oscillations and meditate functional processes.

In monofractal (as opposed to multifractal) analysis, fractal signals are described by a single parameter, the Hurst exponent (H), that reflects the global scaling behavior of a system. H is a measure of the correlation structure in a signal (Eke et al., 2000), where H < 0.5 indicates anticorrelation in the signal, H = 0.5 indicates there is no correlation (it is a random white noise or walk), and H > 0.5 indicates positive correlation or the presence of long-memory in the process. Like many other physiological signals, the BOLD time-series exhibits long-range correlations across a hierarchy of time-scales as shown by consistent findings of H > 0.5 in cortical regions of the brain (Fadili and Bullmore, 2002; von Wegner et al., 2018). In fMRI fractal analysis, higher H values suggest that there is longer memory in the BOLD signal, meaning that past dynamics more strongly mediate future processes in the brain. Conversely, lower H values reflect less correlated fluctuations and a more disordered structure in the BOLD signal. Importantly, the strength of correlation in neural signals largely impacts how the brain is able to process and function. This has been demonstrated in numerous studies that have highlighted the physiological relevance of H in the BOLD signal (Maxim et al., 2005; Wink et al., 2006; Barnes et al., 2009; He, 2011; Lei et al., 2013; Sokunbi et al., 2014; Churchill et al., 2015, 2016; Gentili et al., 2015, 2017; Dong et al., 2018).

BOLD signal H values seem to reflect internal changes in the brain (i.e., aging and disease progression) (Maxim et al., 2005; Sokunbi et al., 2014; Dong et al., 2018). They are also affected by external perturbations to the system as demonstrated by various task-based studies. He (2011) showed that H values in all 21 brain regions analyzed were lower while performing a button-pressing task relative to during resting state conditions (watching cross-hair). This finding of weaker correlation in the BOLD signal during task has been observed in multiple studies (Barnes et al., 2009; Ciuciu et al., 2014; Churchill et al., 2016). Furthermore, task novelty and cognitive effort correlate with reductions in H across multiple task conditions; more novel and demanding tasks predict lower H values (Churchill et al., 2016). In line with these findings, Barnes et al. (2009) showed that task difficulty relates to the recovery time of scale-free dynamics using a rest-task-rest paradigm. It takes more time for the brain to return to its pre-task H value when the preceding task requires more cognitive effort, suggesting that greater cognitive load causes a larger shift from more correlated to less correlated dynamics. In this lower-H task state, the signal is less constrained by a strongly-correlated and redundant signal structure characteristic of the resting state. It has been suggested that this shift in signal dynamics may allow for more efficient processing of new information and better performance on discrete tasks (He, 2011).

In addition to the use of conventional tasks and resting state conditions, researchers are increasingly interested in using more complex, naturalistic conditions to study brain function. Such conditions (e.g., movie-watching, listening to stories, playing video games) are thought to be more ecologically valid, and in some instances, have been shown to impart advantages for fMRI studies (Vanderwal et al., 2015, 2017, 2019; Sonkusare et al., 2019; Eickhoff et al., 2020; Finn and Bandettini, 2021). One motivating idea within naturalistic imaging is that naturalistic conditions evoke neural responses that are unique (Hasson et al., 2010; Nastase et al., 2020). For example, as noted by Hasson et al. (2010), individual neurons in primary visual cortex of anesthetized cats showed different firing rates during naturalistic viewing compared to frequently used conventional task conditions like visual grating stimuli, suggesting that in some brain regions the timing of neural responses may be more precise during naturalistic conditions (Dan et al., 1996; also see Gallant et al., 1998 for similar findings in the macaque). At the BOLD signal level, we know that signal changes during movie-watching are concerted across subjects, and that this synchronized activity covers a large portion of the cortex (Hasson et al., 2004). These intersubject correlations demonstrate predominantly lower frequency bands in temporal and frontal regions, and high frequency bands in visual cortex (Kauppi et al., 2010). Recent dynamic analyses in movie fMRI showed that functional connectivity networks interact and reconfigure into a reliable repertoire of states during movies, and that relative to rest, these brain states are more plentiful and varied (Meer et al., 2020). Using MEG to measure band-limited power during movie-watching, Betti et al. (2013) showed that most resting state networks had a decrease in power during movies, and showed greater variability in power in the visual occipital cortex. They concluded that though the spatial topography of networks was maintained from rest to movies, the frequency domain underwent significant modulation (Betti et al., 2013). In a small sample of healthy adults, multi-scale entropy of the signal overall was greater during movies and rest relative to task (Vanderwal et al., 2019). Together, these findings indicate that naturalistic conditions alter temporal and frequency characteristics of BOLD signal in unique ways, but to date, few papers have attempted to directly assess signal characteristics during movie fMRI compared to conventional eyes-open rest.

In this context, we wanted to determine if the BOLD signal during movie-watching had different fractal dynamics than during rest, whether any cross-condition differences in H values were network specific, and if the test-retest reliability of H-values were different in movies and rest. Based on the findings cited above in which task-processing decreased H-values compared with rest, we hypothesized that movie-watching (as a robust task state) would have lower H values than rest overall, and that this effect would be especially pronounced in networks known to be involved in movie processing (e.g., visual network, frontoparietal network). We further predicted that reliability of H values would be comparable across movies and rest. These hypotheses were tested using the open-source Human Connectome Project (HCP) data 7T release, which had a high sampling rate (TR = 1 s) and many time-points (~900 volumes per condition), as well as both Rest and Movie watching paradigms in the same subjects.

## Methods

### HCP Data

Data for this study are from the Human Connectome Project's Young Adult cohort (HCPS1200 release https://www.humanconnectome.org/) (Van Essen et al., 2012). The resting state and movie-watching runs from the 7T fMRI dataset were used for all analyses.

#### Participants

One thousand two hundred and six healthy young adults were recruited *via* the Missouri Family Registry from 2012 to 2015. The Washington University in St. Louis Institutional Review Board was the approving body for all parts of the Human Connectome Project, and all participants gave written informed consent. Participants scanned at 7T were first recruited as part of the original 3T HCP dataset.

#### 7T fMRI Scanning Procedure

**Figure 1:**
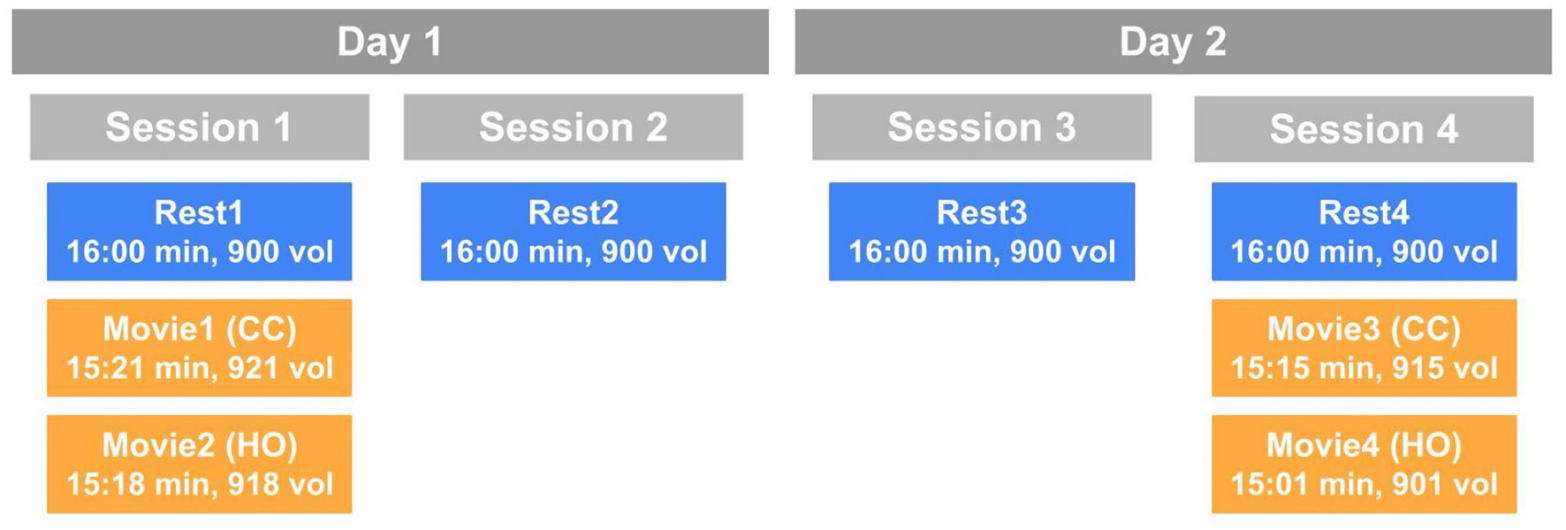
A schematic of the HCP scanning protocol by day and session. Blue boxes indicate resting state runs and orange boxes represent movie-watching runs. CC, Creative commons; HO, Hollywood excerpts.

Subjects were scanned across four sessions and 2 days, resulting in a total of 4 resting state (Rest1, Rest2, Rest3, and Rest4), and 4 movie-watching (Movie1, Movie2, Movie3, Movie4) runs. Session 1 and Session 2 were conducted on Day 1, and Session 3 and Session 4 were conducted on Day 2. One resting state run was acquired at the beginning of all sessions. Movie-watching data were only acquired in Sessions 1 and 4. Subjects watched Movie1 and Movie2 on Day1, and Movie3 and Movie4 on Day4 (Figure 1). Anatomical data were acquired on a 3T machine on a previous date.

#### Resting State Scans

Each resting state run was 16 min long. Subjects' eyes were open and they were asked to fixate on a bright cross-hair projected on a dark background.

#### Movie-Watching Runs

All movie-watching scans are between 15:01 and 15:21 min. Movie1 and Movie3 are a concatenation of independent short clips from Creative Commons (CC) licensing on Vimeo.com (Movie1 example: https://bit.ly/3t7hI7Y). Movie2 and Movie4 are different collections of Hollywood excerpts (HO) published by Cutting et al. (2012). All movies included 20 s of rest between each clip and at the start and end of the full movie. Each movie compilation also included the same repeat validation clip from a CC Vimeo movie (1 min, 24 s). Subjects watched Movie1 and Movie2 following Rest1, and Movie3 and Movie4 following Rest4.

#### Acquisition Details

Structural data (T1 weighted and T2 weighted scans) were collected on a customized Siemens 3T (Connectome Skyra) with a standard 32-channel Siemens head coil. 3T structural (along with fMRI and diffusion MRI acquisitions not used in the current study) were collected over four imaging sessions of ~1 h each. T1 weighted scans were acquired using a 3D MP-RAGE sequence with the following parameters: TR = 2.4 s, TE = 2.14 ms, TI = 1 s, flip angle = 8°, FOV = 224 × 224 mm, matrix size = 320 × 320 mm, slice thickness = 0.7 mm, voxel size = 0.7 × 0.7 × 0.7 mm^3^, image acceleration = 2, scan time = 7 min 40 s. T2 weighted scans were acquired using a 3D T2-SPACE sequence with the following parameters: TR = 3.2 s, TE = 565 ms, FOV = 224 × 224 mm, matrix size = 320 × 320 mm, slice thickness = 0.7 mm, voxel size = 0.7 × 0.7 × 0.7 mm^3^, image acceleration = 2, scan time = 8 min 24 s.

Functional MRI scans were collected using a 7-Tesla Siemen's Magnetom scanner. 7T scanning sessions involved resting state fMRI, movie-watching fMRI, retinotopy fMRI and diffusion MRI acquisitions collected over four imaging sessions of ~1.25 h each (see https://www.humanconnectome.org/storage/app/media/documentation/s1200/HCP_S1200_Release_Reference_Manual.pdf for full details). fMRI gradient-echo EPI runs used the following parameters: TR = 1 s, TE = 22.2 ms, flip angle = 45°, FOV = 208 × 208 mm^2^, matrix size = 130 × 130, 85 slices, slice thickness = 1.6 mm, voxel size = 1.3 × 1.3 × 1.3 mm^3^, multiband factor = 5, image acceleration = 2, scan time per run = ~15 min. Resting state and movie-watching runs were acquired using the same parameters, but the difference in movie durations resulted in a different number of volumes per run. The resting state scans all have 900 time-points, while Movie1, Movie2, Movie3, and Movie4 have 921, 918, 915, and 901 time-points, respectively.

#### Sample

Data from all 184 subjects with 7T fMRI data were downloaded. Participants were excluded if they had a mean framewise displacement (FD) of >0.15 mm in any single run, leaving 143 subjects. Because our central question hinged on cross-condition comparisons, we assessed mean FD across conditions, and found a significant difference (movie < rest). We then ran a simple motion-matching algorithm, removing those participants who had the greatest mean FD from Rest until we had a cohort with no significant difference in mean FD across conditions (MOVIE) (*p* = 0.166, 95% CI [−0.006, 0.001]). The sample used in all analyses going forward comprised 72 participants (41 females; mean 29.46 ± 3.76 standard deviation years of age).

### Preprocessing

The pre-processed ICA-FIX denoised fMRI data from HCP was downloaded and used for this study. According to HCP, independent component analysis (ICA) was run on high-pass filtered scans using FSL's MELODIC (https://web.mit.edu/fsl_v5.0.10/fsl/doc/wiki/MELODIC.html) (Smith et al., 2004; Woolrich et al., 2009; Jenkinson et al., 2012), and FSL's FIX (Griffanti et al., 2014; Salimi-Khorshidi et al., 2014) was subsequently used to identify and remove the artifactual components. Motion parameters were also aggressively regressed out of the data. We applied a 5 mm FWHM smoothing to the cleaned data.

### BOLD Measures

Preprocessed ICA-FIX denoised data were smoothed with a 5 mm FWHM kernel using the fslmaths program and run through AFNI's 3dRSFC program (https://afni.nimh.nih.gov/pub/dist/doc/program_help/3dRSFC.html) (Taylor and Saad, 2013) to generate ALFF maps for each subject and condition. ALFF was calculated after 0.01–0.08 Hz bandpass filtering. The standard deviation of the BOLD signal was also calculated for the whole brain voxel-wise.

### Fractal Analysis

There are many different methods used to calculate H depending on the domain (i.e., frequency or time) and signal class of the data. A power-spectrum based method (frequency domain) was used to calculate H, as this approach has been shown to be sensitive to both tissue type and activation in fMRI data (Rubin et al., 2013). The power spectrum of all gray matter voxels was calculated using Welch's periodogram (using the python library SciPy.Signal) with eight windows of 50% overlap on a restricted frequency range of the data (above 0.01 Hz), mirroring the parameters used by Rubin et al. (2013). PSD plots of gray-matter voxels were generated to confirm power-law scaling across frequencies >0.01 Hz (Supplementary Figure 1). β was calculated as the negative slope of a straight line fitting the PSD distribution on a log-log scale.

The Beta value of a signal determines its signal class. Generally, a fractal process is realization of one of two classes: fractional Gaussian noise (fGn), defined as β < 1, or fractional Brownian noise (fBm), defined as β > 1 (Eke et al., 2000). fGn signals are stationary processes with constant variance, whereas fBm signals are non-stationary processes with increasing variance over time. As detailed by Eke et al. (2000), the calculation of H depends on signal class, where H is related to β $H=\frac{\left(\beta +1\right)}{2}$ and $H=\frac{\left(\beta -1\right)}{2}$ for fGn and fBm signals, respectively (Eke et al., 2000). This method generates standard values between 0 and 1 for both signal classes. While this interpretation of H (0 < H < 1) is commonly used in the literature, it bears confusion in the field as it does not reflect the signal's class. Processes with the same H value that are of a different class will have β values differing by 2 and thus very different scaling properties. This leads to erroneous fractal estimates and ambiguity in interpretations. Alternatively, the concept of “extended H″ (H′), where 0 < H′ < 2, reflects the signal class of the data. Here, 0 < H′ < 1 describe fGn processes and 1 < H′ < 2 describe fBm processes (Eke et al., 2000; Hartmann et al., 2013). In efforts to mitigate uncertainty and confusion, H′ values are used in this analysis, but are henceforth simply referred to as H. The extended Hurst exponent was calculated as $H^{\prime }=\frac{\left(\beta +1\right)}{2}$. In-house Python scripts used for calculating H are available on our lab's GitHub: https://github.com/WeberLab/FractalDimension/blob/master/welch.py.

### ROI Analysis

Gray matter and 7 functional connectivity-based network masks were made using FSL. Yeo 2011 7-network “liberal” RSN masks were downloaded from FreeSurfer (https://surfer.nmr.mgh.harvard.edu/fswiki/CorticalParcellation_Yeo2011), cropped and resampled to the HCP's volumetric functional MNI space using FSL's FLIRT utility (Jenkinson and Smith, 2001; Jenkinson et al., 2002).

Gray matter ROI was created by thresholding FSL's MNI avg152 tissueprior gray, white and CSF probabilistic maps to above 50%, subtracting remaining white and CSF voxels from the gray, and resampling to HCP dimensions as above. The networks, as identified in Yeo et al. (2011), are: visual (Vis), somatomotor (SoM), dorsal attention (DAtt), ventral attention (VAtt), limbic (Lim), frontoparietal (FP), and default network (DN) (Yeo et al., 2011).

### Reliability

The intraclass correlation coefficient (ICC) was used to measure and interpret the test-retest reliability of H during movie-watching and rest. A two-way random, absolute agreement model with single measures was used [commonly known as ICC(2,1)] (Noble et al., 2021). The ICC of H was calculated for Movie (where all 4 movie runs are raters) and for Rest (where all 4 resting state runs are raters) to determine the stability of H within each condition.

### Investigating Fractal Origins: Motion and Movie Features

The fractal properties of head movement and the movie stimuli were analyzed in order to investigate if fractal patterns in the brain are driven by intrinsic (e.g., neuronal) or extrinsic (e.g., motion) factors.

#### Motion

The H value of every subject's FD time-series was calculated using the same fractal analysis method described above.

#### Movie Stimulus

The time-series of three movie features [brightness, zero-crossing rate (ZCR), and root mean square (RMS)] were extracted from each movie to analyze visual and auditory temporal dynamics. This was done using Pliers, a Python package for automated feature extraction of multimodal stimuli (http://psychoinformaticslab.github.io/pliers/index.html). For all four movies, the H value was calculated for each feature.

### Statistical Analysis

All statistical analyses were conducted using RStudio (R Studio Team, 2020). H was compared between Rest and Movie in the gray matter using a paired Student's *T*-test for all 8 regions of interest (gray matter and 7 networks). Multiple comparisons among the networks were corrected for using Holm's Step-Down Procedure (Holm, 1979). In order to compare ICC values between conditions, Rest and Movie ICC values were calculated with bootstrapped resampled data 1,000 times. The 95% confidence intervals (CIs) of the difference in ICC values were then calculated using the percentile method.

## Results

### Whole-Brain H Is Greater in Movie Than Rest

**Figure 2:**
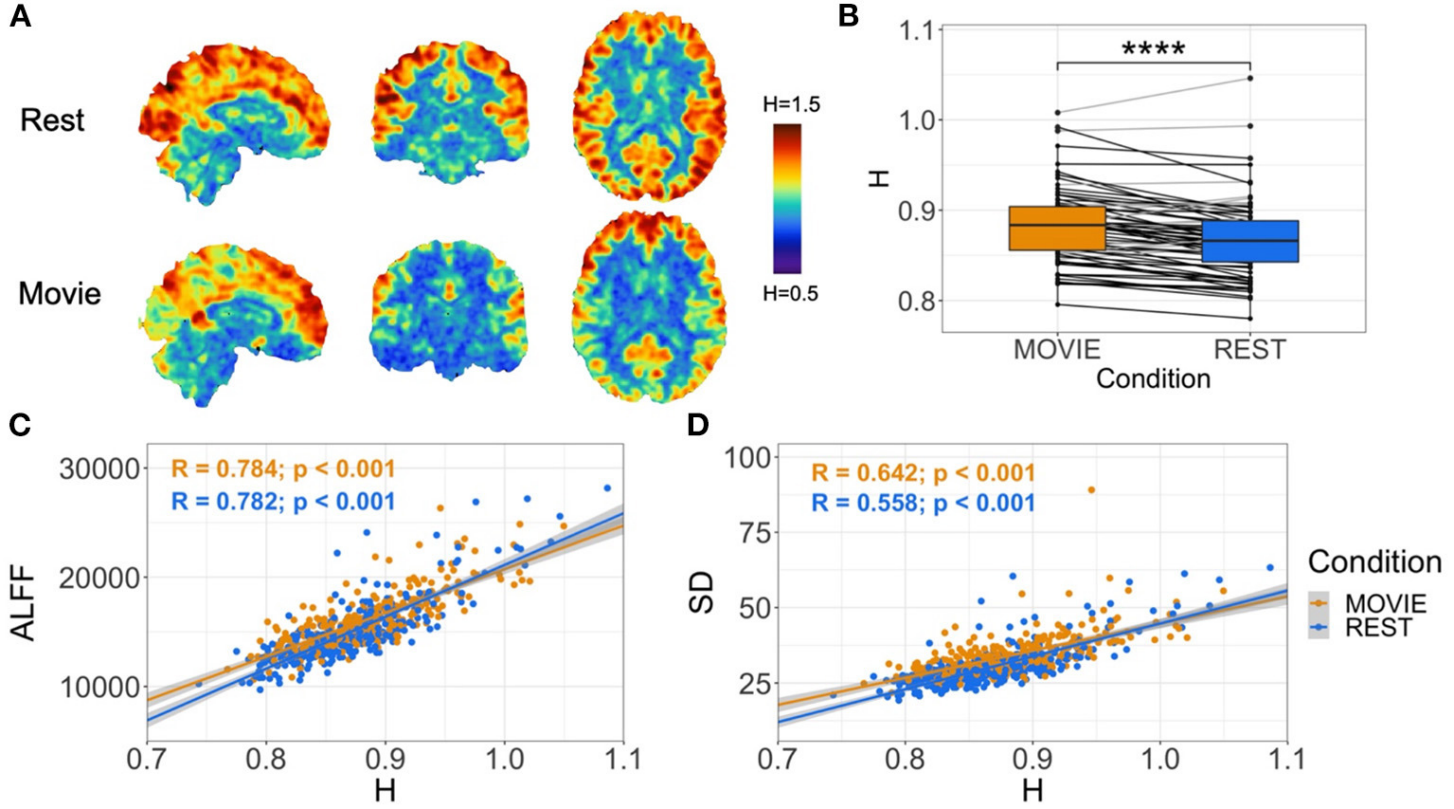
Cross-condition comparison of whole-brain H (*N* = 72). **(A)** H values in rest (top) and movie-watching (bottom) of sample sagittal, coronal and axial slices in a single subject. Images show distinction between cortical gray and white matter, and values that appear greater in Movie relative to Rest in a non-homogeneous distribution. **(B)** Box-plot of H between Movie Watching (left, orange) and Rest (right, blue) showing group-level finding that H-values are significantly greater in Movie. Black lines depict participants with greater H in Movie than Rest, and light-gray lines depict participants with lower H in Movie than Rest. **(C,D)** Scatter-plot of H vs. ALFF and H vs. standard deviation of BOLD values, respectively, with Movie (orange) and Rest (blue), showing positive correlations for both conditions in both cases. Gray shadows over orange and blue lines depict the 95% confidence interval for predictions from a linear model.

Contrary to our original hypothesis, movie-watching resulted in significantly higher H values in whole-brain gray matter (Figure 2A; mean difference = 0.014; *p* = 5.279 × 10^−7^; 95% CI [0.009, 0.019]). This means that overall there is more persistent fractal phenomena and scale-invariant behavior in the brain while watching a movie relative to rest. The effect size is small (Cohen's D = 0.32), with an increase in H observed in 54 subjects and a decrease in 18 (Figure 2B). H in both conditions is positively correlated with amplitude of low frequency fluctuation (ALFF) (Movie: r = 0.784; *p* < 0.001; Rest: r = 0.782, *p* < 0.001) (Figure 2C) and standard deviation (SD) (Movie: r = 0.642; *p* < 0.001; Rest: r = 0.558, *p* < 0.001) of the BOLD signal (Figure 2D).

### Cross-Condition Changes in H Are Network-Specific

**Figure 3:**
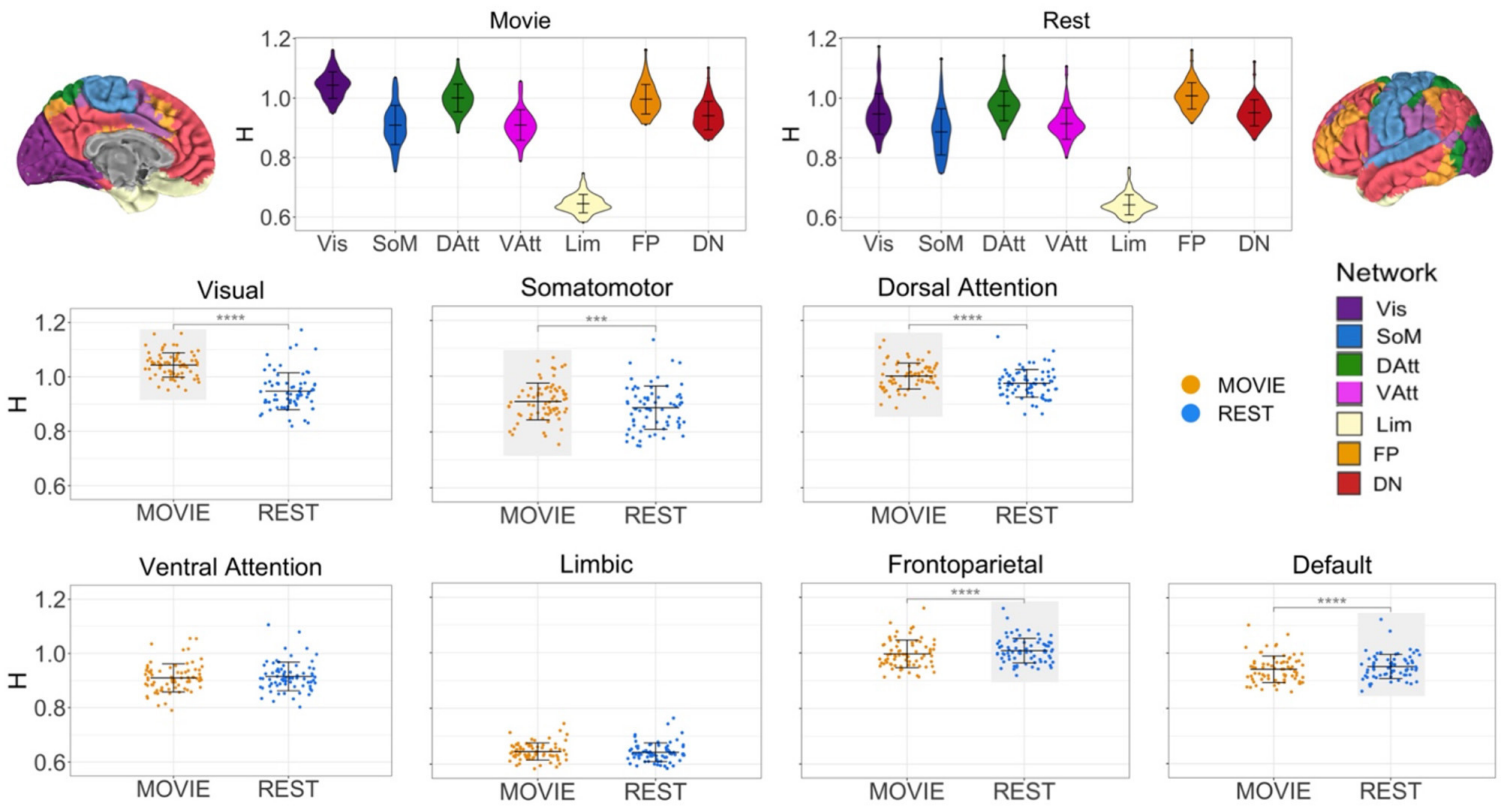
H across conditions, by intrinsic connectivity network (*N* = 72). Top center: violin plots for Movie and Rest showing H values of seven resting state networks (RSNs) from Yeo et al. Brains at the top left and right show topography of those networks using the same colors. H values ranged from 0.69 to 1.24, suggesting that BOLD signal within all networks demonstrates self-similarity. Bottom: scatter plots of H by condition for each RSN separately. Horizontal lines show the mean and standard deviation of H. Light gray boxes highlight which condition (Movie or Rest) had the higher statistically significant average value. Three networks had higher H values during Movie (visual, somatomotor and dorsal attention networks), and two networks had higher H values during Rest (frontoparietal and default networks).

While at the whole-brain level H is greater during movie-watching, the cross-condition comparisons differ between networks (Figure 3). H is significantly higher in Movie than Rest in the visual (mean difference = 0.096, adj-*p* = 2.11 × 10^−25^, 95% CI [0.084, 0.108]), sensorimotor (mean difference = 0.022, adj-*p* = 1.21 × 10^−4^, 95% CI [0.113, 0.033]), and dorsal attention (mean difference = 0.026, adj-*p* = 1.1 × 10^−14^, 95% CI [0.021, 0.003]) networks. Conversely, H is significantly lower during movie-watching in the frontoparietal (mean difference = −0.012, adj-*p* = 9.74 × 10^−6^, 95% CI [−0.017, −0.007]) and default (mean difference = −0.010, adj-*p* = 3.15 × 10^−5^, 95% CI [−0.014, −0.005]) networks. There is no significant difference across conditions in the ventral attention (adj-*p* = 0.054, 95% CI [−0.010, 8.08 × 10^−5^]) and limbic (adj-*p* = 0.167, 95% CI [−0.001, 0.007]) networks.

### Reliability of H in Movie and Rest Condition

**Figure 4:**
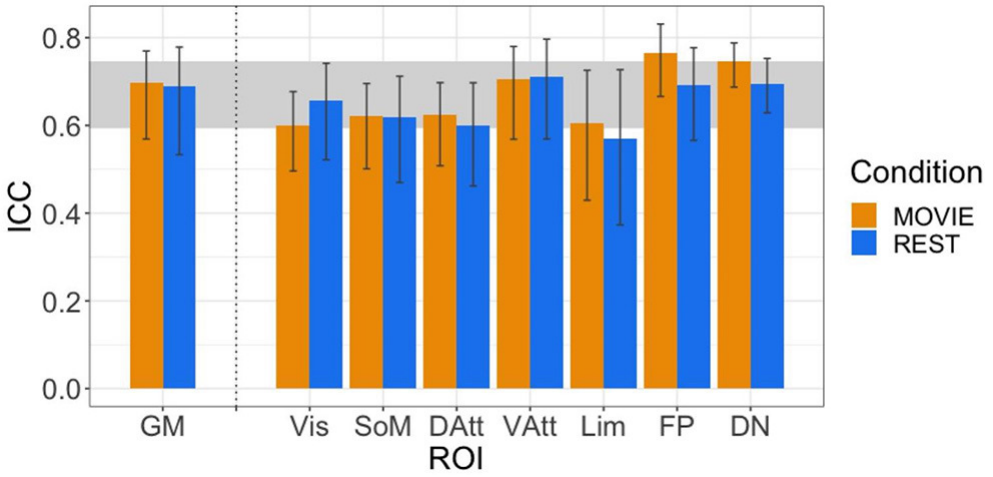
ICCs of H across resting state runs and different movies. GM shown on left, with the seven RSNs on the right (separated by vertical dashed line). Vertical black bars represent 95% confidence intervals. Horizontal gray band in background represents good test-retest reliability (0.60 < ICC < 0.74).

The ICC values during movie-watching were 0.698 (95% CI [0.569, 0.770]) and 0.688 during resting state in the gray matter (95% CI [0.533, 0.779]) (Figure 4). ICC values are interpreted as: poor <0.4, fair 0.4–0.59, good 0.6–0.74, and excellent > 0.75 (Noble et al., 2021). Overall in the gray matter, the test-retest reliability of H is good in both conditions. It is also good across most of the networks, with exceptions being the limbic network during rest (fair reliability) and the frontoparietal network during movie-watching (excellent reliability). The 95% CI of the difference between gray matter Rest and Movie ICC is [−0.137, 0.180], using 1,000 bootstraps of the original samples. Since the interval includes 0, the difference in reliability is not significant.

### Investigation of Fractal Origins

**Figure 5:**
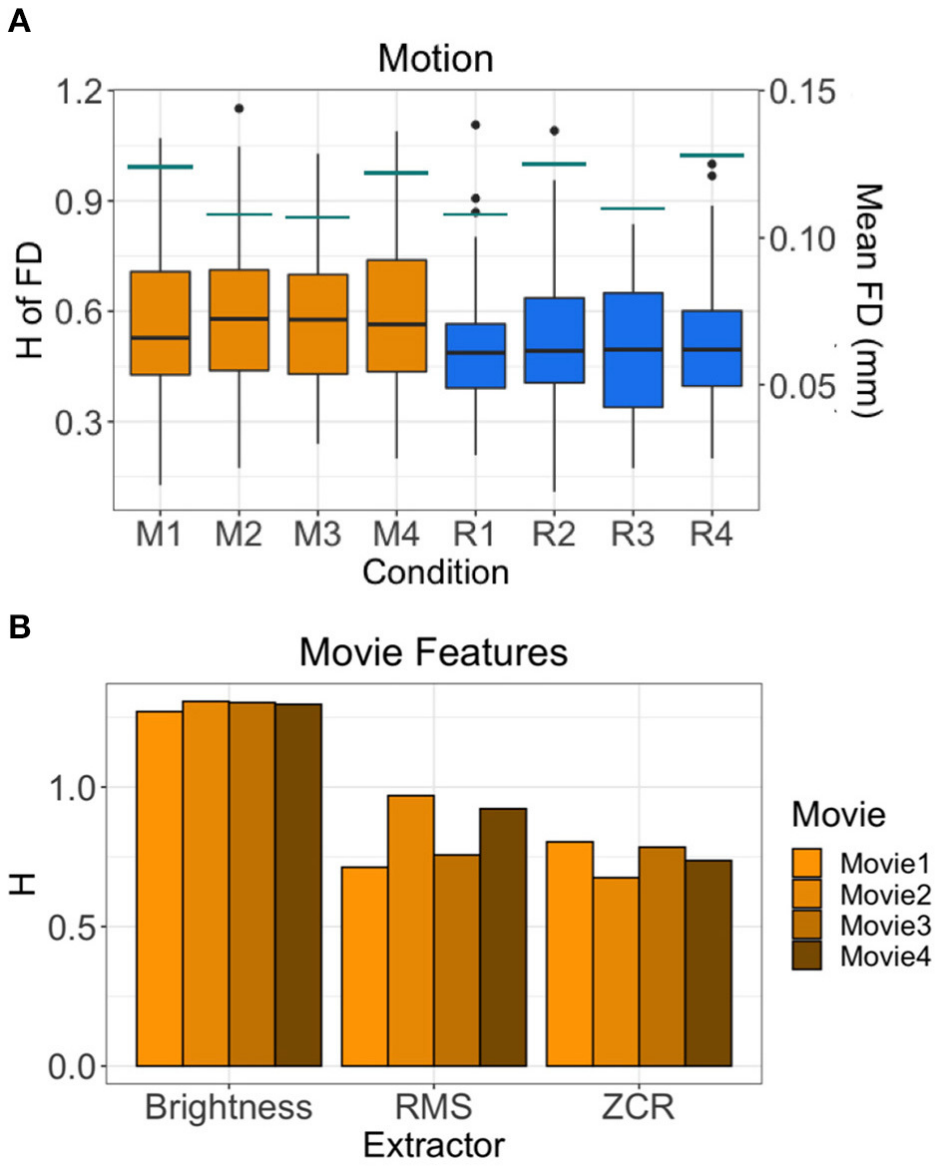
H values of other possible sources of fractality. **(A)** H values of the FD time-series of all 72 subjects by run are shown in the boxplots. Orange boxplots are the Movie runs (M1 = Movie1, M2 = Movie2, M3 = Movie3, M4 = Movie4), blue are the Rest runs (R1 = Rest1, R2 = Rest2, R3 = Rest3, R4 = Rest4). The mean FD values are shown by the horizontal teal lines for each run using the secondary y-axis (right). **(B)** H values of the time-series of three movie features (brightness, RMS, ZCR) for each movie.

The average H of the FD time-series are between 0.494 and 0.602 (Figure 5A). This suggests that the motion of subjects has weaker fractal properties and is a less correlated, more random temporal process. Across all runs, the H value of FD time-series during movie-watching is significantly greater than rest (mean difference = 0.077; *p* = 1.709 × 10^−10^; 95% CI [0.054, 0.100]). The mean FD for each run is between 0.107 and 0.128 mm (Figure 5A). Of the movie features, the brightness time-series has the highest mean H across movies (1.294 ± 0.016), followed by RMS (0.840 ± 0.125), then ZCR (0.750 ± 0.057) (Figure 5B).

## Discussion

### Whole-Brain H Values

In this study, we found that fMRI BOLD data demonstrates scale-invariant behavior during both movie and resting state conditions. Spatially, the whole-brain voxel-wise H values qualitatively differentiated between tissue type (i.e., higher values in gray matter and lower values in white matter and cerebrospinal fluid regions) and demonstrated a non-uniform pattern of distribution that aligns with functional specialization (e.g., higher values in visual cortex during movies and lower values in primary sensory and motor cortex during rest, Figure 2). Additionally, to aid in the assessment of the H values, we calculated the standard deviation (SD) and the amplitude of low-frequency fluctuations (ALFF) of the BOLD signal for each voxel. In the gray matter, for both movie and rest, we found that H is positively correlated (r ~ 0.7) with both SD and ALFF, which is also consistent with previous studies (Churchill et al., 2016; Gentili et al., 2017). These positive relationships suggest that scale-invariant phenomena relate to known measures of variability, and are more prominent in regions with greater neurovascular variability and lower frequencies (Akhrif et al., 2018), respectively. Overall, the range of H values in these 7T data, their spatial distribution, and their positive relationship to SD and ALFF are all in keeping with previous findings of H in BOLD signal during resting state conditions (Wink et al., 2008; He, 2011; Herman et al., 2011; Churchill et al., 2016; Gentili et al., 2017). The novel contribution is that we provide the first analysis of H in naturalistic movie-watching conditions, with over 900 volumes of data per subject per condition across 4 independent runs.

### Gray Matter ROI

In the whole-brain gray matter mask, we found that H values were higher during movie-watching than the resting state. This means that movie-watching induces a more temporally redundant, autocorrelated signal structure than exists during rest. In other words, during movie-watching there is stronger long-range dependence in the signal such that past dynamics more heavily mediate future brain processes. During the resting state, on the other hand, the BOLD signal is less positively correlated and more disorder is present in the underlying dynamics. This is in marked contrast to our original hypothesis based on previous task-based fractal findings, which found that H decreases from rest during a task (Barnes et al., 2009; He, 2011; Churchill et al., 2015, 2016). The differing findings suggest that the fractal dynamics involved in signal processing may depend on the nature of the stimulus. Here, we compared the processing of two different stimuli: a movie, which is continuous and multimodal, and a cross-hair, which is stationary and not very ecologically-valid. By quantifying the difference in fractal dynamics during the processing of each stimulus, our findings reveal novel information about the possible signal mechanisms involved in naturalistic perception and the function of scale-free dynamics in processing.

Mechanistically, the scale-invariant properties during movie-watching may emerge to help support the continuous perception of the movie. When processing a movie the brain must dynamically reconfigure to keep integrating the continuously changing sensory information. Recent investigations have found that the brain becomes “multistable” while movie-watching (Meer et al., 2020), meaning that the brain switches between weakly stable states during processing. In a multistable system, each state is defined by an independent attractor and the jump from one attractor to the next is driven by noise processes (Freyer et al., 2012). Using the hidden Markov model, Meer et al. found that during movie-watching, the brain transitions through a higher number of brain states (ten) than in the conventional resting state (two), and that the state shifts occur at a faster rate (Meer et al., 2020). When interpreting these brain-state findings with our signal-based findings, we speculate that as the brain organizes and reorganizes to assemble the higher number of brain states while watching a movie (Meer et al., 2020), the underlying BOLD signal develops a more strongly correlated, self-similar signal structure to support the transitions. Our whole-brain findings suggest that fractal processes may be a key mediator of continuous perception by driving neural activity through a sequence of state transitions that help predict and update brain states more efficiently.

Fractal dynamics may emerge to support the state transitions during movie-watching because it is functionally advantageous when processing a continuous stimulus. A system that has weak multi-stability, maximal dynamic range, and no dominant temporal scale is thought to be capable of the most efficient responses to the widest array of events (Kardan et al., 2020b). With greater flexibility across a hierarchy of time scales, the brain can easily transition between attractors and quickly adapt based on the demands of the environment. As higher H values reflect closer proximity to this state (Kardan et al., 2020a), our findings suggest that movie-watching evokes fractal dynamics that better mediate optimal processing than the conventional resting state. Recent findings support this theory, which found that higher H in individuals predicts greater improvements on an audio-visual dual n-back task (Kardan et al., 2020b). The authors suggest that subjects in a higher-H state process tasks and transfer information more efficiently when operating near criticality, leading to greater performance improvements. Therefore, our finding supports the idea that movie-watching evokes a state of optimal neural functioning that may better reflect the endogenous state of the brain than a fixed cross-hair.

### Network-Level Results

It is important to note that while the difference in H between movie and rest conditions in the gray matter is statistically significant, it was not observed within every subject and it is not large (Cohen's D = 0.32/small). This may be due to the bidirectional changes in H observed across networks; H is significantly higher during movie-watching in three networks and significantly lower in two. While this may have led to a smaller effect overall in the gray matter, it provides some new information about the temporal structure of BOLD signal in these networks during naturalistic viewing. In line with previous studies reporting network-specific fractal parameters (He, 2011; Ciuciu et al., 2014), these data suggest that individual networks exhibit distinct temporal dynamics that depend on the demands of the current task or acquisition state.

A main finding of this work is the network-level differences in H between Movie and Rest. During movie-watching, the networks with significantly higher fractal parameters compared to rest were the visual, somatomotor, and dorsal attention networks, and the networks with greater H in rest were the default and frontoparietal networks. As this is the first investigation of fractal properties under naturalistic conditions, it is difficult to make inferences about the reason for this “three up, two down” pattern. Of the three that increased, we will focus our discussion on the visual network, where the movie > rest effect for H was most pronounced. Unsurprisingly, the visual network is known to be highly involved in movie processing, with intersubject correlations covering much of the occipital cortex during most movies (Hasson et al., 2004; Golland et al., 2007). Kauppi et al. showed that ISCs in visual regions occur at higher frequency bands than those in frontal and temporal regions (Kauppi et al., 2010), and as mentioned in the introduction, Betti et al. used fMRI and MEG to show greater variability in power of the visual network signal during movies relative to rest. Regions known to process various aspects of movies are contained within the visual network [e.g., V1–V3 for luminance changes, V5+, V3A, medial posterior parietal cortex and lateral occipital cortex for local motion processing (Bartels et al., 2008)], and recent work by Owen et al. indicate that when analyses are geared toward identifying higher-order dynamics, visual regions are highly involved even during story-listening (i.e., visual regions support narrative processing even in the absence of visual input). In line with recent work, we also suggest that aspects of higher H values in any region may pertain to non-cognitive neural processes, such as the generation of noise or off-line homeostatic processes that may be part of this active, dynamic state (Laumann and Snyder, 2021). Here, we underscore the clear finding that signal fractality in the visual network is markedly greater during movie-watching relative to eyes-open rest, and suggest that this may be due to a combination of first- and second-order processes–and possibly also non-cognitive processes–that it either orchestrates or participates in during movie-watching.

Conversely, H was significantly greater during resting state than movie-watching in two higher-order networks: the frontoparietal (FP) and default networks (DN). These cross-condition differences were small but significant, and they held true at the individual subject level. Task-based studies suggest that the frontoparietal network functions as “a flexible hub of cognitive control” (Marek and Dosenbach, 2018). It is thought to control behavior in a goal-driven and flexible manner by interacting with other networks to a uniquely high degree during difficult cognitive tasks (Cole et al., 2013; Cocuzza et al., 2020). Previous movie-based work indicates that FC of the frontoparietal network (*via* dual regression) is higher during movies relative to rest (Vanderwal et al., 2015). More specifically, a unique study that included resting state, a finger-tapping task, and a movie-watching condition showed that the frontoparietal network exhibited connectivity patterns during movies that were in fact the opposite of those observed during rest, such that during movie, the FP became positively correlated with the DN (Gao and Lin, 2011). Similarly, Caldinelli and Cusack recently showed that though the frontoparietal network demonstrated the most reconfiguration during conventional task conditions, it was not the most flexible hub during movie-watching (Caldinelli and Cusack, 2021). These studies suggest that FP function is state dependent, and observations about its function during task or rest do not extend to movie-watching. The lower fractal parameter observed here also suggests a unique state-dependent signal structure within this network relative to rest, and future work is needed to better understand FP function during movie-watching, and to relate more interpretable measures of FP function to H.

A similar set of observations and questions relate to the default network finding of lower H during movie relative to rest. The DN is classically thought of as being most active when not engaged in a task or focused on the internal environment. It supports a myriad of cognitive processes including mind-wandering and self-referential processing, and conventional task and resting state situations, is most often anti-correlated with task-positive or frontoparietal regions (Anticevic et al., 2012; Raichle, 2015; Buckner and DiNicola, 2019). Recent work has shown that the DN plays an important role in naturalistic processing. For example, when connectome-based predictive modeling was used to classify a connectome as coming from rest or movie conditions, the DN contributed the second highest number of parcels (after visual network) to that differentiation (Sanchez-Alonso et al., 2021). Multiple studies have shown that the DN supports the processing of, and possibly the memory of, longer narratives such as those present in movies and stories (Lerner et al., 2011; Simony et al., 2016; Baldassano et al., 2017; Tikka et al., 2018; Nguyen et al., 2019). A recent dynamic intersubject functional connectivity analysis of the DN during movie-watching identified strong correlations with the experience of surprise, but not for other events (e.g., events with high theory of mind, emotional intensity or perceived importance) (Brandman et al., 2021). The authors interpreted this surprise-effect as being an instantiation of the much broader phenomenon of predictive error-related processing (Clark, 2013; Pine et al., 2018), and in particular, of the interplay that underlies ongoing narrative comprehension and updating. This internal/external updating interplay might also be consistent with the high default network connectivity previously observed during the *Inscapes* paradigm, in which participants have to use internal and previously made observations to interpret and process abstract shapes and imagery (Vanderwal et al., 2015). Again, much work is still needed to more fully characterize default network functioning during movie-watching, particularly as some of the observations made using task data do not seem to apply to movie-watching.

Here we show that the self-similarity of the BOLD signal for both the default and frontoparietal networks remains fractal during movie-watching, but that the fractality is lower during movie than rest. Together, this “three up, two down” pattern suggests that networks engaged by more primary processing of stimuli become more fractal during movies, whereas heteromodal cortex appears to achieve greater fractality at rest. Perhaps there is something about the cognitive dynamic processes involved in movie-watching that shifts these higher-order networks to a slightly less efficient or optimal–or less “natural”—state. For example, movie-watching entails an inherently passive observership in which one can engage in high-level social processing with absolute certainty that one will not have to respond or have any agency in the unfolding events (Redcay and Schilbach, 2019; Lee et al., 2020). This passive observership may, roughly speaking, “turn off” parts of these networks, or diminish ways in which they interact with other regions. In this model, these networks would become even more fractal if we were able to measure BOLD signal in more truly interactive and naturalistic conditions. A second possibility is that these two higher-order networks have evolved to perform “optimally” or to be at their most fractal or “metastable” state at rest, seemingly as part of the brain's ability to be continuously responsive and reactive in the face of ongoing stimulation and dynamic inputs. In this model, H values for these networks would always be highest during rest, and differing states or processing demands would modulate that fractality to varying degrees. Currently, these are loosely held conceptual questions, and it is not clear how the observed cross-condition shifts in H relate to neural processes or to a state of “optimal” processing or readiness.

### Reliability

When we computed ICCs of H values across four resting state runs and four movie watching runs, we obtained values of ~0.7, indicating good reliability. Interestingly, these measures contain between-session variability (i.e., runs from different days), which in the case of functional connectivity estimates, has been shown to have more impact on functional reliability than between-condition reliability (O'Connor et al., 2017). The confidence interval of the difference between bootstrapped movie and rest ICC values crosses zero, so we conclude that ICCs of H are similar for both rest and movie conditions. One caveat is that while the ICCs for rest represent a fair estimate of test-retest reliability, the ICCs for movie are computed across runs that used different movies, providing an estimate of cross-movie consistency rather than actual test-retest reliability. These results are similar to other reliability assessments that have focused on movie-rest comparisons. For example, using the same dataset, Tian et al. showed that functional connectivity (FC) measures across the different movies were similar to those across resting state runs (Tian et al., 2021). Overall, our findings indicate that H can be computed with good reliability using both movies and resting state conditions, despite the use of different movies and the acquisition of scans on different days.

### H of Other “In-Scanner” Factors

We wanted to test whether in-scanner head motion might be fractal, as prior work has shown that human movement such as gait (Hausdorff, 2007) and finger-tapping (Coey et al., 2015) exhibit fractal characteristics. The resulting H-values for mean framewise displacement time-courses were near 0.5, and we therefore conclude that head motion in this sample was not overtly fractal at this coarse level. Other work has also shown that movies themselves exhibit fractal properties. For example, Cutting et al. (who contributed to the movie stimuli used in the HCP 7T scanning) summarize how shot length, scene duration, motion changes, and sound amplitude all exhibit high degrees of self-similarity (Cutting et al., 2010, 2018). They attribute this to the filmmaking process in which the film is crafted to fit or to be congruent with endogenous human attentional processes (Gilden, 2001, 2009; Shimamura et al., 2015). Here, we extracted statistical time-courses of visual and auditory features of the movie, and show that even in isolation, these time-courses demonstrate self-similarity. The high H values of the extracted movie features further support the idea that movies evoke ecologically-valid dynamics since scale-invariant, hierarchical processes have more similar statistical properties to the real world (Sonkusare et al., 2019; Meer et al., 2020). Within this tautological framework, it becomes exceedingly difficult to assess whether BOLD signal during movie-watching is itself inherently fractal or whether it is more fractal because it is evoked by fractal stimulation or signals. For example, is the significant effect of H in the visual network discussed above driven in whole or in part by fractal visual input? Future work is needed to properly test and understand these relationships.

### Limitations

The overarching finding of this study is that BOLD signal reliably demonstrates fractal properties during both movie-watching and resting state conditions. As Cutting et al. succinctly state, “Perhaps we should assume that fractality (Stadnitski, 2012) is the null hypothesis when considering naturally or socially occurring, complex temporal or spatial structure” (Cutting et al., 2018). In contrast, previous work has clearly shown that H values decrease significantly during conventional scientific tasks (Barnes et al., 2009; He, 2011; Churchill et al., 2016). Given that there are multiple ways to calculate H and a lack of standardization when performing fractal analysis, it is challenging to directly compare previous findings in the field to ours. Therefore, a major limitation in the current work is that the dataset did not have a task condition with which to compare the movie and rest findings. We speculate that if all three conditions were included in the same study, H would be greatest in the movie, then rest, then task conditions. Another limitation is that the movie watching runs in the HCP data include multiple 20 s epochs of resting state which we did not remove because we did not want to violate the signal dynamics within a run for these particular signal processing analyses. Consequently, it is possible that the cross-condition differences in H observed here are actually an underestimation. Third, the movies used in the HCP study are arbitrary and highly specific stimuli. There are particularities about the movies that could result in movie-specific findings that might not generalize, for example, to a horror film or a cartoon. Even both Hollywood compilations contain movies from different eras (Movie2: 2001–2020, Movie4: 1980–2000) and therefore contain significant differences in scene length, shot duration, and many other technical features (Cutting et al., 2010, 2018) that could differentially influence BOLD-signal dynamics. Finally, we used resting state networks as a meaningful measure of functional organization in the brain, but these networks likely privilege resting state. Future studies might reveal important differences in fractality across states using movie-derived parcellations (e.g., Bottenhorn et al., 2018).

## Conclusion

We investigated the difference between the brain's fractal dynamics during movie-watching and eyes-open resting state conditions using 7T fMRI data. In the gray matter overall, we found that H values were higher in the movie condition, suggesting that movie-watching evokes more scale-invariant and positively correlated dynamics in the BOLD signal. At the network-level, H was greater during movie-watching in the visual, somatomotor, and dorsal attention networks, and this effect was especially robust in the visual network. Interestingly, we found that H values in higher order networks (frontoparietal and default) showed the opposite effect, with slightly higher H values at rest. The test-retest reliability of H was overall “good” in both conditions, providing evidence that H can be measured even across different movies with comparable reliability to that attained across resting state runs. These findings provide new information about BOLD signal characteristics during naturalistic movie watching conditions, and open up new questions about the purpose and source of scale-invariant dynamics during naturalistic conditions. In particular, we suggest that the overall fractality observed during both movie-watching and rest sheds new light on the lower H BOLD signal patterns previously observed during conventional tasks. Future work might investigate the shifts between fractal and non-fractal patterns of signal within a region or network, and might assess the relationship between a region's functional characteristics (e.g., connectivity, modularity, or multistability) and signal self-similarity.

## Data Availability Statement

The original contributions presented in the study are included in the article/Supplementary Materials, further inquiries can be directed to the corresponding author.

## Ethics Statement

The studies involving human participants were reviewed and approved by the Institutional Review Boards (IRB) of Washington University in St. Louis, MO, United States (IRB # 20120436). The patients/participants provided their written informed consent to participate in this study.

## Author Contributions

TV conceived the main question. TV and AW designed the analyses and performed additional statistical analysis. OC contributed data and analysis tools, performed the analysis, and wrote the paper with major contributions from TV. OC, TV, and AW contributed to editing and rewrites. AW contributed funds from grants. All authors contributed to the article and approved the submitted version.

## Funding

The authors of this study were financially supported by the British Columbia Children's Hospital Research Institute (Establishment Award and Salaries). Data were provided by the Human Connectome Project, WU-Minn Consortium (Principal Investigators: David Van Essen and Kamil Ugurbil; 1U54MH091657) funded by the 16 NIH Institutes and Centers that support the NIH Blueprint for Neuroscience Research.

## Conflict of Interest

The authors declare that the research was conducted in the absence of any commercial or financial relationships that could be construed as a potential conflict of interest.

## Publisher's Note

All claims expressed in this article are solely those of the authors and do not necessarily represent those of their affiliated organizations, or those of the publisher, the editors and the reviewers. Any product that may be evaluated in this article, or claim that may be made by its manufacturer, is not guaranteed or endorsed by the publisher.

## Supplementary Material

Supplementary Figure 1 Power spectral density plots for a single subject. The blue lines show the average power across grey matter voxels vs. frequency on a log-log scale during rest **(A)** and movie **(B)**. The orange line is the slope of the linear regression.
