# Supplementary figures and images for: Fractal-Based Analysis of fMRI BOLD Signal During Naturalistic Viewing Conditions
Source: Front Physiol. 2022 Jan 11;12:809943. doi: 10.3389/fphys.2021.809943 (PMC8787275; doi:10.3389/fphys.2021.809943)

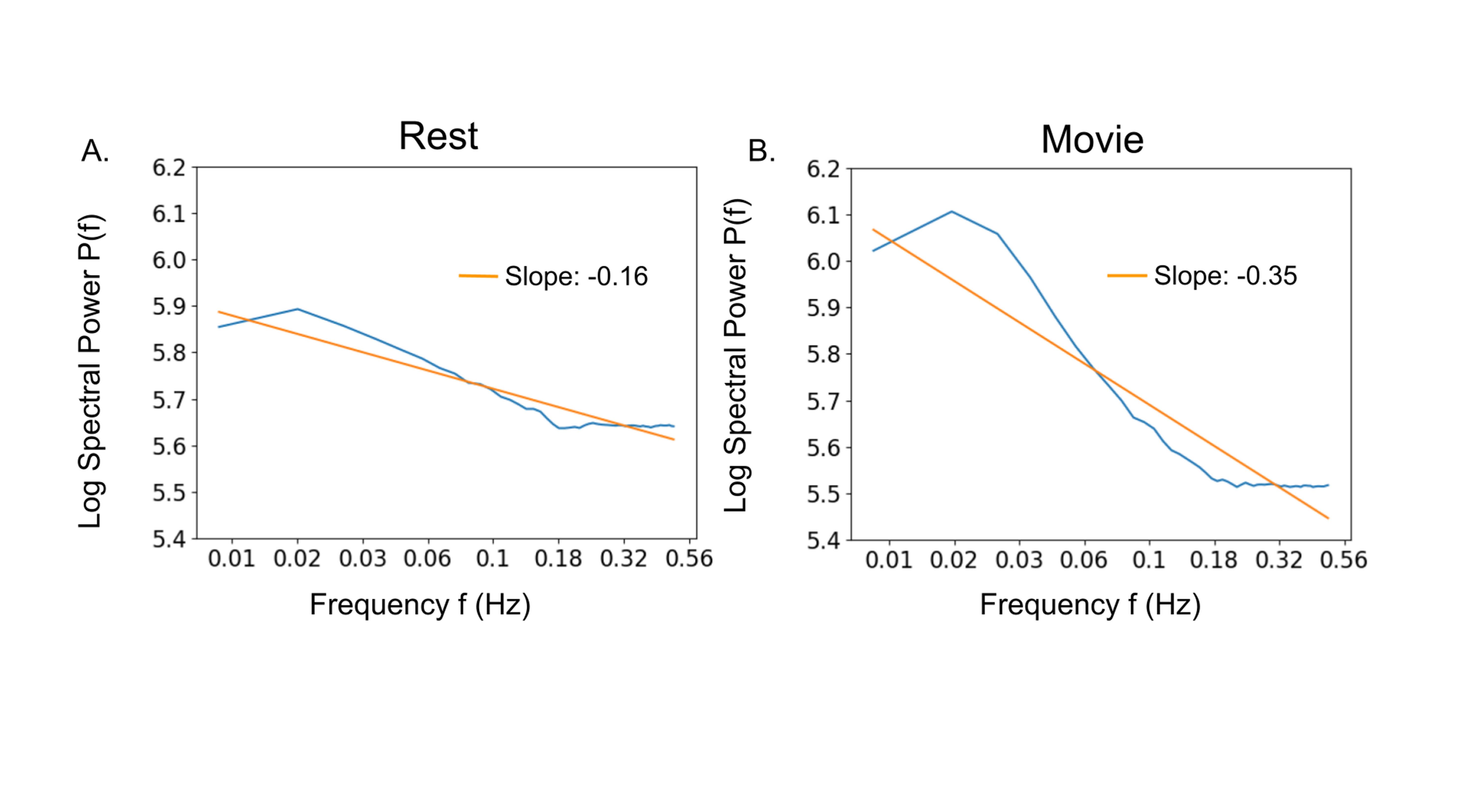

Supplement: Supplementary Figure 1 — Power spectral density plots for a single subject. The blue lines show the average power across grey matter voxels vs. frequency on a log-log scale during rest (A) and movie (B). The orange line is the slope of the linear regression. [file Image_1.TIFF]
